# Endothelial cells and their role in the vasculature: Past, present and future

**DOI:** 10.3389/fcell.2022.994133

**Published:** 2022-09-15

**Authors:** Ramani Ramchandran

**Affiliations:** Department of Pediatrics, Division of Neonatology, Children’s Research Institute, Children’s Wisconsin, Medical College of Wisconsin, Milwaukee, WI, United States

**Keywords:** vessel, cilia, angiogenesis, signaling, VEGF, tip cell, stalk cell

## Introduction

The circulatory system is critical for the development of the vertebrate organism and is comprised of an intricate network of blood and lymphatic vessels. Vessels consist of an inner lining of endothelial cells (EC or ECs) that are in contact with the blood or lymph and an outer lining of smooth muscle cells that surrounds the endothelium. In this specialty Grand Challenge article, I will discuss the past, present and future in the field of EC biology, a topic of my interest and expertise. The intent here is to provide my perspective of how the EC biology field has progressed in the last 40–50 years. My discussion here is restricted to EC biology and interaction of ECs with other cell types in a contextual manner. The purpose of this Grand Challenge article is to inform the reader of the exciting developments that have come and yet to come in the EC biology field, and to invite fellow scientists to contribute their innovative work to the Molecular and Cellular Pathology Section of the Frontiers in Cell and Developmental Biology journal.

## The past

ECs are specified from precursor cells called angioblasts that emerge from the lateral plate mesoderm of a developing embryo. Angioblasts differentiate into venous or arterial lineage early in development, and later, lymphatic ECs emerge from an established vein. This process of *de novo* angioblast differentiation into arteries and veins is also referred to as artery vein specification and occurs through vasculogenesis, a process involving assembly of cells to form a tube-like structure called a vessel. This vessel has a central hollow cavity called a lumen, which carries fluid such as blood or lymph. In contrast to vasculogenesis, angiogenesis is the process of sprouting blood vessels from an established vasculature and is often associated with remodeling. We have gained immense insight in the last century or so on how vessels are patterned, which has largely built upon the initial careful descriptions of vessel architecture in various organs noted by the Belgian physician Andreas Vesalius among others. The discovery of growth factors in particular vascular endothelial growth factor (VEGF) or vascular permeability factor (VPF) by Dvorak (Dvorak et al., 1979; Senger et al., 1983) and Ferrara (Ferrara and Henzel, 1989) groups, and their cognate receptors (Millauer et al., 1993; Fong et al., 1995; Shalaby et al., 1995) opened the field of ECs biology from descriptive events to mechanisms that help explain how ECs emerge and form vessels. Further, the discovery of nitric oxide (NO) a gas from ECs that regulates function of other cells provided additional mechanistic insights in the field. This discovery was awarded the Nobel Prize in Physiology or Medicine in 1998. In terms of VEGF, knockout of a single *VEGF-A* allele in mice caused embryonic lethality (Ferrara et al., 1996) and abnormal vessel development, which highlighted the importance of this factor in vascular development. Further, hypoxia, a state of low oxygen, which is often observed in a growing vessel bed induces VEGF expression to promote angiogenesis (Shweiki et al., 1992; Mukhopadhyay et al., 1995). However, how hypoxia induced VEGF expression was not clear. The breakthrough discovery that hypoxia induces transcription factors (hypoxia induced transcriptional factor HIF-1, HIF-2) (Forsythe et al., 1996; Ivan et al., 2001) that binds to specific regions in the *VEGF* promoter to induce its expression provided the answer (Shweiki et al., 1992). Soon, various splice isoforms of VEGF-A (VEGF-A_189_, VEGF-A_165_, VEGF-A_144,_ VEGF-A_121_) (Park et al., 1993), other ligand members in the family (VEGF-B, VEGF-C (Kukk et al., 1996), PDGF, FGF, PlGF), and primary receptors such as VEGFR-1, VEGFR-2, and co-receptors Neuropilin-1 and -2 were discovered. These molecules and their overlapping expression patterns in the developing and adult vasculature highlighted the complexity of the vascular system. It also became clear that vessel patterning was a sophisticated and highly controlled process. One intriguing observation that was previously described in anatomy books but gained new meaning was the striking resemblance between nerve and vessel patterning (Carmeliet and Tessier-Lavigne, 2005). These two structures from a morphological anatomical perspective lay side-by-side, and from a molecular mechanism perspective, share ligands and receptors. These similarities posed the obvious question as to whether crosstalk occurred between these systems. Indeed, the identification and investigation into the role of axon guidance molecules (Slits/Robos, Ephrin/Eph, Semaphorin/Plexins and Notch/Delta) and their function in the developing vasculature provided answers to the crosstalk question. The emerging theme from these studies were that vessel sprouting or inhibition was context dependent and involved a delicate balance of ligand-receptor signaling output. Until recently, ECs were thought to be a relatively homogenous in terms of structure and function, with only a few exceptions namely artery and venous ECs that show distinct transcriptional signature and function. However, concepts from the axon guidance and vessel guidance fields suggested that perhaps, all ECs in a growing sprout are not the same. The Betsholtz group in 2003 (Gerhardt et al., 2003) demonstrated in an elegant paper that indeed the retinal ECs at the growing tip were vastly different than the following stalk cell in that they were non-proliferative and only sprouting in nature. The stalk cell that followed the tip cells were proliferative and helped extend the growing sprout. Subsequent work identified phalanx cells, cells that are quiescent and behind the stalk cells in an established vasculature. The discovery that all ECs in a growing sprout were not the same led to an astonishing number of studies in a short time period that partly defined the mechanism that underlie this specification process. Delta-Notch and VEGF-VEGFR2 ligand-receptor signaling systems emerged as key players in the process, and more molecules were soon shown to contribute to the tip/stalk cell specification mechanism (Hellstrom et al., 2007; Siekmann and Lawson, 2007; Suchting et al., 2007; Jakobsson et al., 2009; Jakobsson et al., 2010; Krueger et al., 2011). At present, the signaling paradigm is far from fully revealed and is an area of active investigation in the field.

As developmental biologists continued to unravel how these molecules worked together to generate new EC types and eventually pattern the vasculature, some of this knowledge helped gain better understanding of vascular anomalies, a condition that is thought to result from deregulated mechanisms underlying embryonic vascular patterning process. Classification of vascular anomalies into two classes: hemangiomas and vascular malformations occurred, and the genetic basis for vascular anomalies gained steam (Chaft et al., 2003; Marler and Mulliken, 2005). Meanwhile, some in the field started to focus on the adult vasculature, mostly from a therapy perspective. The major emphasis of the adult angiogenesis studies was perhaps in three areas of medicine namely tumor biology, retinal biology and cardiovascular biology. In tumor biology, the concept of blocking vessel growth (i.e., to prevent angiogenesis) so that tumors can be starved of blood supply for their growth seemed liked a promising idea. A host of approaches ranging from anti-angiogenic growth factors, ligand blocking antibodies, receptor blocking antibodies, small molecule inhibitors and aptamers were developed to target tumor growth (Ferrara and Kerbel, 2005). However, many of these approaches failed in the clinic (Jain, 2014). A positive side effect of the ligand blocking strategies especially those targeting VEGF in the tumor field turned out to be extremely beneficial in the treatment of the age-related macular degeneration of the wet kind in the eye field (Barakat and Kaiser, 2009). Avastin (anti-VEGF, Genentech) and Pegaptanib (Anti-VEGF, Macugen) along with two other Anti-VEGF drugs clearly offer benefit to these patients and has been a gamechanger in this field. Patients treated with these antibodies are able to read the eye chart, which they previously could not. In cardiovascular medicine, the ambitious goal of single injection of VEGF in a gene therapy setting to restore functional collateral arteries for blocked arteries turned out to be much more challenging than expected. Such setbacks in the application of ECs knowledge in the clinic directed the field to return to the basics of ECs biology.

## The present

Over the last decade or so, basic ECs biology has seen some remarkable progress. We now have a great understanding of the various types of junctions that exist between ECs. VE-cadherin plays a prominent role in stabilizing the vascular junctions (Lampugnani et al., 1995). We also know that ECs from different vascular beds express different kinds of junctional proteins and have tighter (brain) or more permeable (liver) junctions depending on the organ they reside in. The role for various isoforms of VEGF-A ligand have been discovered, and specific effects for these isoforms in retinal and other vascular beds have been identified. We also know in quite intricate detail how VEGF signals through its receptors to trigger specific phosphorylation events that result in readouts of proliferation, migration, apoptosis, endothelial tube formation, junctional stability, permeability and angiogenesis. The reader is directed to specific reviews on VEGF signaling pathway in ECs (Claesson-Welsh, 2016) for a detailed understanding of these cascades and the key amino acid residues on the receptors that trigger specific signals. The trafficking of VEGFR2 inside the EC and how this is mediated through the classical endosome, clathrin-coated or caveolin vesicular pathways have been identified (Salikhova et al., 2008; Jopling et al., 2009; Lanahan et al., 2010; Ballmer-Hofer et al., 2011; Simons, 2012; Fearnley et al., 2016). Although VEGF:VEGFR2 was considered as a paradigm for ligand-cell surface receptor signaling, this concept needed revisiting given the identification of ligand-independent non canonical autocrine VEGF signaling in ECs (Lee et al., 2007). Thus, our understanding of EC signaling in a context specific fashion has grown leaps and bounds, and there is more to come.

In the last decade, the concept of metabolism in endothelium and how it influences tip, stalk and phalanx cells started to become clearer. The tip versus stalk cell differentiation was previously thought exclusively to be a genetic signal mediated process. The Carmeliet group discovered that VEGFR2 signaling in the tip cells increases the expression of 6-phosphofructo-2-kinase/fructose-2,6-bisphosphatase3 (PFKB3) (De Bock et al., 2013), that regulates glycolysis used by ECs for ATP production. These glycolytic enzymes are located in the filopodia and lamellipodia regions, regions of high metabolic activity allowing for rapid ATP production and need for actin cytoskeletal remodeling to promote migration of tip cells. ECs line the lumen and have plenty of access to oxygen and thus would be predicted to utilize oxidative metabolism for their energy. Interestingly, ECs are glucose addictive, and generate 85% of their ATP *via* glycolysis even though glycolysis only yields two molecules of ATP per molecule of glucose. This behavior is reminiscent of cancer cells displaying Warburg effect, the concept of metabolizing glucose in anerobic rather than aerobic conditions. This anerobic glucose metabolism offers an advantage to ECs in their function in that they do not use the oxygen but allow it to diffuse into the needy avascular anoxic tissue. This evolutionary adaptation and possible implications in disease are beginning to emerge (Fraisl et al., 2009; Verdegem et al., 2014).

ECs are also the first line of contact with blood flowing in the lumen. Thus, the role of flow in regulating endothelial cell morphology emerged. ECs experience fluid shear stress, which is the tangential force (blood flow) exerted on the endothelium. In response to shear stress in culture, some ECs such as human umbilical vein ECs align to the direction of flow while others such as brain ECs do not (DeStefano et al., 2017). Fluid shear stress also triggers activation of specific transcription factors (KLF family) (SenBanerjee et al., 2004; Wang et al., 2010) that induce gene expression and signaling pathways to trigger changes at the cell junctions. However, the question of how ECs sense shear stress to transduce signals inside remains unclear. This concept of mechanotransduction, which is the conversion of mechanical stimulus to electrochemical activity in relation to ECs was poorly understood. A microtubule-based organelle called primary cilia (Goetz and Anderson, 2010) expressed on apical side of EC was shown to be important for transducing shear stress into biochemical calcium signals in ECs (Hierck et al., 2008; Nauli et al., 2008; Egorova et al., 2012; Ando and Yamamoto, 2013). Endothelial cilia were also shown to bend to a particular angle *in vivo* to low shear stress and release calcium *in vivo* in a zebrafish model (Goetz et al., 2014). Further, we showed with confocal analysis in zebrafish embryos that cilia were observed prior to flow on assembling ECs suggesting a role for cilia in vasculogenesis (Eisa-Beygi et al., 2018). We and others (Kallakuri et al., 2015) have reported that endothelial cilia are required in a cell autonomous fashion for brain vascular integrity. Thus, endothelial cilia role in vascular barrier function is an emerging field (Ma and Zhou, 2020; Thirugnanam et al., 2022). More recently, primary cilia have also been observed in lymphatics and surprisingly was noticed in the abluminal side (Paulson et al., 2021), which suggests additional novel functions for cilia that remains to be discovered. In terms of luminal versus abluminal side of the blood endothelium, the concept of polarity of ECs during vascular development in embryo and adult and the underlying mechanisms responsible for establishing and maintaining polarity has been revealed. ECs are polarized in that the surface that faces the blood or lumen is called apical side, and the side that faces the tissue or matrix is referred to as abluminal or basolateral. Significant insights into EC polarity have occurred through study of lumen formation during angiogenesis, and the effect of shear stress on ECs (Li et al., 2002; Koh et al., 2008; Tomar et al., 2009; Zovein et al., 2010; Xu et al., 2011; Kwon et al., 2016). In both cases, intracellular signaling molecules of the Rho GTPase family, in particular Cdc42, Rac and Rho and their effectors play a key role in determining the polarity of ECs. There are many proteins involved in cell polarity which is not possible to mention here, and the reader is pointed to reviews in that field (Koh et al., 2008; Bowers et al., 2016). From a VEGF signaling perspective, the two main tyrosine kinase signaling receptors namely VEGFR1 and VEGFR2 have been identified to differentially locate to the lumen and abluminal side respectively in the brain and retinal microvasculature but not the lung (Hudson et al., 2014) vessel bed. VEGF-A triggered signaling through luminal VEGFR1 led to AKT activation, while VEGF-A triggered signaling through abluminal VEGFR2 to increase permeability *via* p38. These studies begin to suggest that ECs respond to the same ligand from two different sides (tissue-derived versus blood-derived) and have vastly different outcomes in terms of their responses.

The emergence of single cell RNA (scRNA) sequencing has revolutionized the field of ECs heterogeneity. It is now increasingly clear that ECs from distinct vascular beds are different and contain sub-sets of cells expressing unique gene expression patterns. For example, 15 distinct cell sub-types have been identified in mouse brain ECs, and similarly, 17 distinct cell types have been identified in mouse lung ECs (He et al., 2018). Further, most ECs mimic the environment that they reside in. In select cases such as adipose- and heart-specific ECs, they overlap in the expression patterns with ECs from other tissues (Paik et al., 2020). This diversity of ECs population suggests that ECs cross talk with a tissue and the cells within it at a specific time and place will be important to determine. Additional insights gained from scRNA studies (Kalucka et al., 2020) include capillary ECs in a tissue show the greatest heterogeneity in gene expression pattern compared to arterial, venous or lymphatic ECs. In some RNA seq studies, similarity in gene expression was noticed between tip and aortic ECs. For example, CXCR4 receptor, a tip cell marker (Strasser et al., 2010) was observed in brain-derived ECs while its ligand CXCL12 is enriched in the arterial EC cluster. These unexpected results provide new questions and directions. We also discovered novel gene regulatory mechanisms in the EC field, which are facilitated by noncoding RNAs. Small and long non-coding RNAs for various vascular expressed genes were identified (Miano and Long, 2015). Our group discovered long non-coding RNAs in *DLL4* and *TIE1* gene loci (Chowdhury et al., 2019). The list continues to grow, and how these noncoding RNAs work together with their protein complexes and other non-coding RNAs is a subject of active investigation in the field.

## The future

The future for EC biology is full of interesting questions and we have the technology to explore them. I think we are entering the golden era of vascular biology from an endothelial cell perspective. The interaction of ECs with resident cells in a tissue is likely to gain steam. With advent of organoid model systems (Scalise et al., 2021), such questions can be addressed in a constructive fashion. Signaling studies in ECs will move into the 3D platform, and integration of flow, circadian rhythm and parameters that make experiments closer to “real-life” will occur. Although my commentary here focused on VEGF signaling for the most part given the abundance of knowledge that we have collectively accumulated, there are many other signaling pathways that have been discovered such as Wnt signaling in ECs that will become integrated with VEGF signaling and others. In this regards, computational and molecular simulation approaches is likely to provide robust hypothesis for experimental tests at the bench. Interdisciplinary collaborative approaches will be needed and is expected to occur more frequently. EC biology that requires more deeper understanding include the role of intracellular organelles such as golgi, mitochondria and others in the context of EC polarity, metabolism, and function. From a therapy perspective, the EC-immune cell interface has many pressing questions and is just beginning to be explored. From my own work, I posit that cilia in endothelium and how they influence EC function is expected to reveal novel insights. Whether cilia from different ECs beds are distinct and serve different purposes are not known. With recent findings from our group that cilia proteins may serve as biomarkers of altered vascular flow (Gupta et al., 2022) opens the door for therapeutic application of this technology. From a molecular biology perspective, long non-coding RNAs, microRNAs, circular RNAs and extracellular vesicle biology in ECs will require integration and more in-depth probing. Ciliary vesicles and its role in paracrine signaling and cargo content will need additional investigation in both normal physiology and disease context. From a physiology perspective, integration of molecular and biochemical events in the context of whole-body physiology will occur. Essentially, the grand challenge here is that we have quite a few Lego pieces to the puzzle and will need to put together some pieces to make sense of the puzzle, and also provide newer pieces to the puzzle for future generations to explore and piece them together. All this effort will culminate in a better robust understanding of how an EC works and functions in a given organ, and how this function may have gone awry in diseases so that we can fix it.
